# A new teaching method of direct fundoscopy for daily clinical practice

**DOI:** 10.1016/j.clinsp.2026.101085

**Published:** 2026-09-05

**Authors:** Aline Shiokawa, Mario L.R. Monteiro, Raffael Massuda, Marcelo Carriello, Ariane B. Fiorentin, Max V. Schramm, Mario T. Sato

**Affiliations:** a Universidade Federal do Parana (UFPR), Curitiba, Brazil; b Department of Ophthalmology and Otolaryngology, Faculdade de Medicina da Universidade de São Paulo, São Paulo, SP, Brazil; c Department of Psychiatry, Universidade Federal do Parana (UFPR), Curitiba, Brazil; d Department of Ophthalmology, Universidade Federal do Parana (UFPR), Curitiba, Brazil; e Universidade Federal do Parana, Brazil

**Keywords:** Ophthalmoscopy, Fundoscopy, Medical education, Clinical practice, Teaching technique, Learning

## Abstract

**Introduction:**

Direct Fundoscopy (DF) is a vital clinical skill used in ophthalmology, neurology, and emergency care. Despite its importance, many medical students graduate without mastering DF, often due to limited practical training and insufficient understanding of ophthalmoscope use. This study proposes a teaching methodology, incorporating theoretical instruction, ophthalmoscope practice, and peer-to-peer simulated examinations in small groups, to enhance students' understanding of direct ophthalmoscopy and improve their ability to apply this knowledge in clinical settings. The aim of this study is to describe an effective methodology for teaching DF, fostering the development of confident and competent future physicians capable of performing this essential examination.

**Material and methods:**

Medical students, divided into groups of five, participated in a supervised DF training period that included both theoretical and practical components. The instruction covered ophthalmoscope operation, proper positioning relative to the patient, and techniques for visualization of the posterior pole of the eye. Students first practiced the complete examination procedure using a poster, followed by peer-to-peer examinations to build confidence. Questionnaires were administered before and after the training to assess students' competence in performing direct fundoscopy.

**Results:**

Following the training, the percentage of students able to visualize any structure of the eye fundus significantly increased from 68.5% to 98.6% (p < 0.0001). Similarly, significant improvements were observed in the visualization of all major retinal structures (p < 0.0001), with the exception of the fovea (p = 0.62). Students also reported a marked increase in their confidence in performing the examination.

**Conclusion:**

The implementation of a standardized teaching methodology was associated with improvement in skills for performing DF when applied to medical students previosly naive to the method.

## Highlights

• Direct Fundoscopy (DF) is a critical clinical skill in many medical fields.
• DF is often underused due to limited practical training during medical education.
• There is a need for a teaching method that improves physicians´ confidence and skill in performing DF.
• We propose a teaching method consisted of a one-day session in 4 steps, combining theoretical and practical components.
• The methodology was associated with enhancement in learning of how to perform DF with more confidence.

## Graphical abstract

**Figure:**
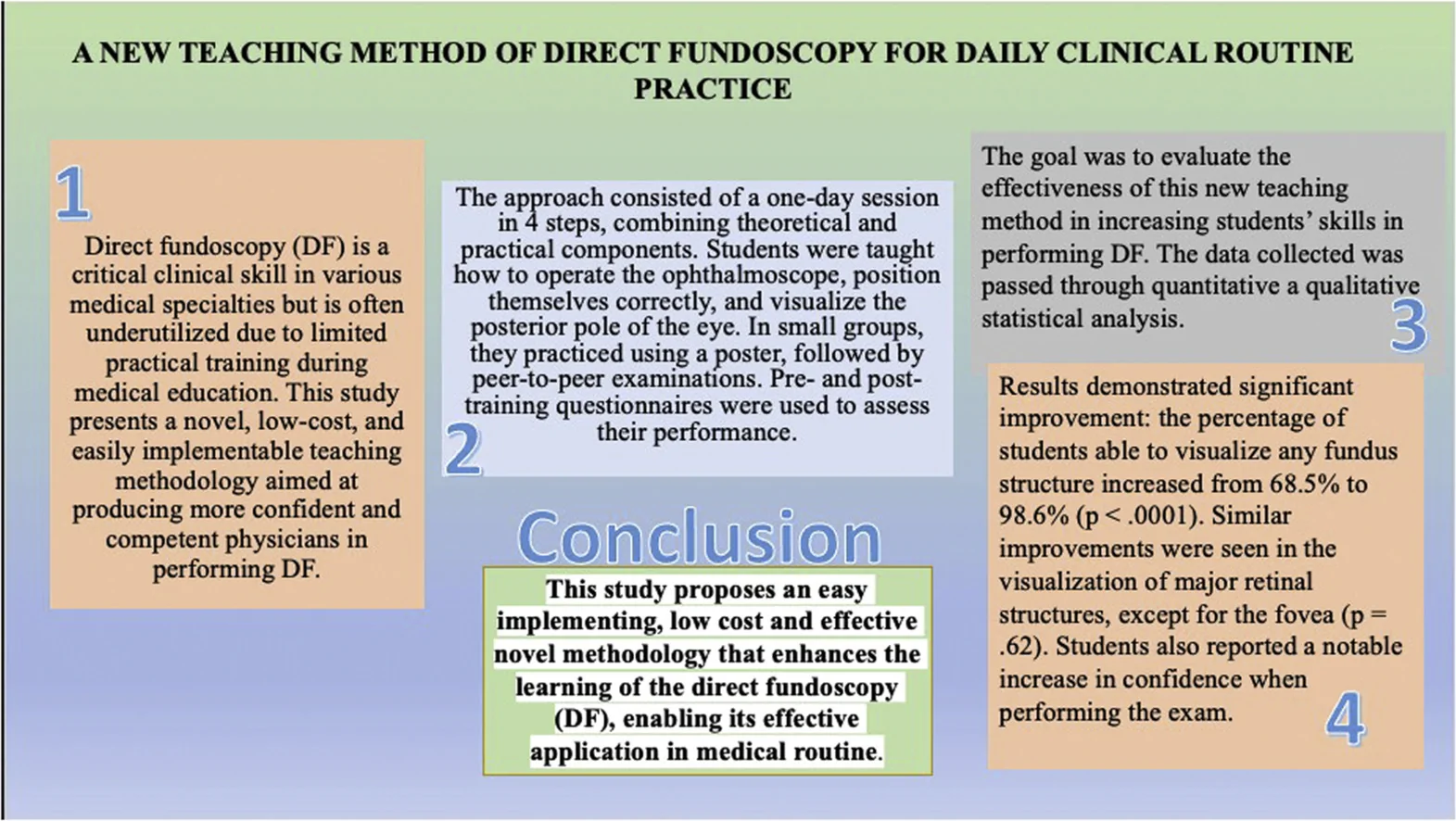

## Introduction

Approximately 4% of clinical visits are related to eye problems.^1^ Direct Fundoscopy (DF), performed with a Direct Ophthalmoscope (DO), is a core clinical skill^2^ that delivers portable, relatively low-cost and wide available tool for ocular assessment, as a part of general physical examination, even for critical care patients.^3^ Direct fundus examination has been the standard exam for evaluating and diagnosing retinopathies for over a century.^4^ However, the emergence of various technologies and imaging techniques has partially or entirely replaced traditional DF. It is important to note that many of these alternative methods, unlike conventional DF through non-dilated pupils, often require costly equipment or extensive patient preparation, potentially complicating routine clinical workflows.

The proficient use of DF is vital in routine medical practice, not only for ophthalmologists but also for general practitioners.^2^ DF is essential for neurological triage during emergencies, enabling observation of the optic nerve for signs of intracranial hypertension.^5^ Furthermore, it is utilized in the evaluation of systemic conditions, such as diabetes, hypertension, and certain rheumatological diseases, including Systemic Lupus Erythematosus.^6^ Additionally, it helps investigate congenital Retinoblastoma, the main intraocular tumor in childhood.^7^

Despite its importance, according to the American Academy of Ophthalmology, less than 50% of medical graduates in the United States of America can perform DF properly.^8^ In 1991, Roberts et al.^6^ reported that less than 5% of doctors routinely used the DF due to a lack of confidence, and that the younger the doctor, the less they used it. This highlights a persistent gap between the DF skills that medical students should acquire and their actual competence in clinical practice. The issue is further compounded by the decreasing emphasis on ophthalmology in medical school curricula, with some universities marginalizing it.^2^

Among the main difficulties faced by students in learning DF are insufficient instruction during practical classes^9^ and a lack of confidence in using the ophthalmoscope.^8^

While newer technologies like mannequins and prototype eyeballs have been introduced for medical education,^10^, ^11^, ^12^, ^13^, ^14^ they often fail to replicate the experience of examining a live patient and may not be readily available, particularly in developing countries. Moreover, studies employing these technologies often lack detailed descriptions of the specific steps involved in performing DF correctly.

Research suggests that students achieve better outcomes when provided with a formal introduction to ocular pathologies, followed by hands-on practice in small groups of five or fewer.^9^ To address the current underutilization of direct fundus examination due to lack of confidence and inadequate training, it is essential to implement innovative learning methodologies that enhance skills and increase the frequency of DF applications when necessary.

This paper aims to present a novel DF teaching methodology, that incorporates a step-by-step technique to enhance the learning of this essential clinical skill. This approach can be readily integrated into medical school curricula with minimal resources, and it has the potential to improve students' confidence in performing DF, ensuring its effective use whenever needed.

## Material and methods

This novel pedagogical approach was implemented in 143 students during the ninth semester of undergraduate medical program at the Federal University of Paraná (UFPR), Brazil. The study was conducted in accordance with the ethical principles outlined in the Declaration of Helsinki, and all participants provided written informed consent prior to their inclusion. The training was delivered by an ophthalmologist instructor to small groups of students, each comprising no more than five individuals. Students completed a questionnaire before and after the training to assess their baseline knowledge of DF, their level of confidence in performing the examination, and the specific challenges they encountered during the practical sessions.

**Fig. 1:**
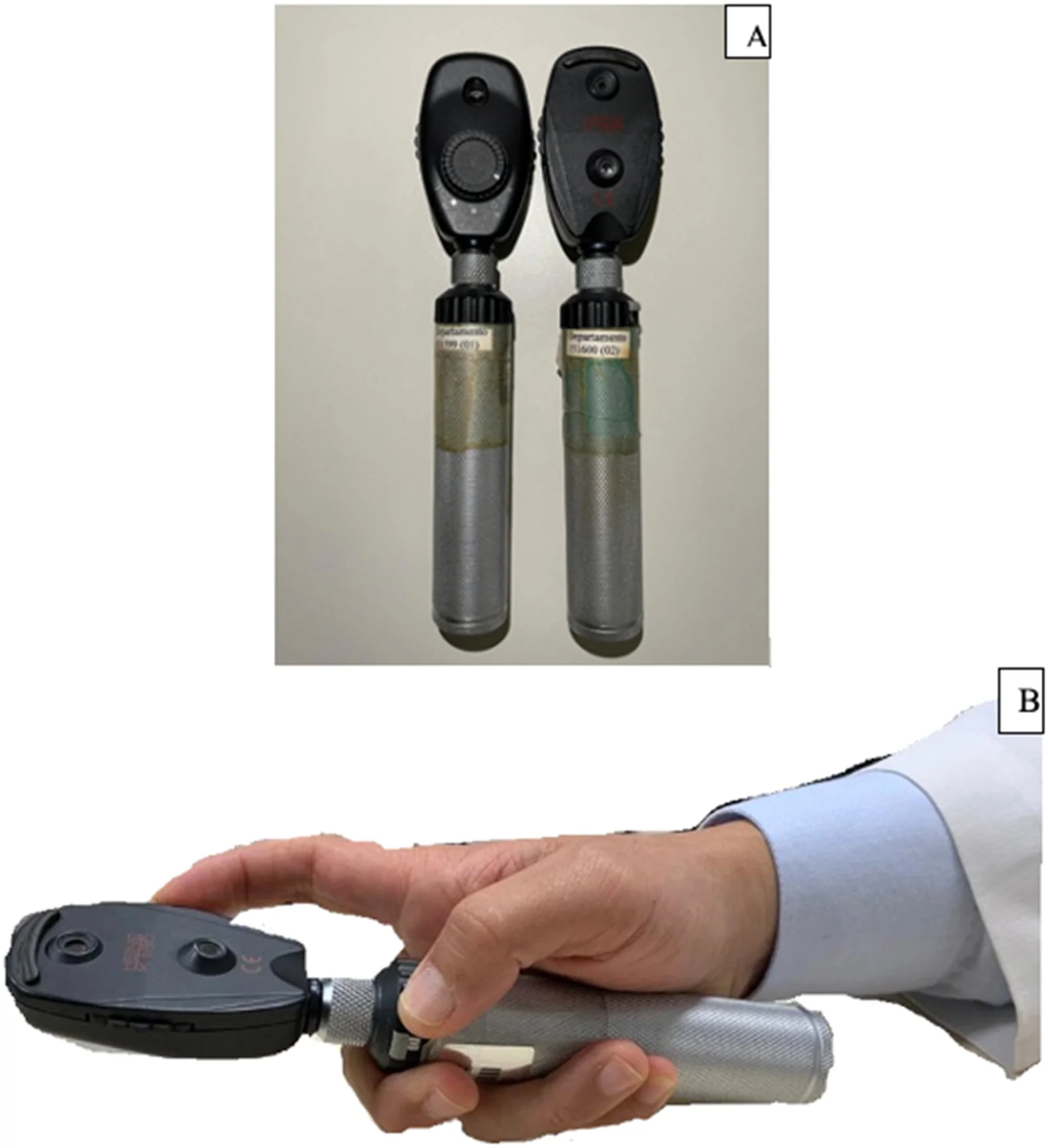
(A) The Heine K-180 ophthalmoscope, left side back view, right side front. (B) How to hold the ophthalmoscope. Source: The authors, 2022.

In this study, the HEINE K-180 direct ophthalmoscope was used. The device, powered by two 1.5V C-size batteries, consists of an ophthalmoscope head and handle (Fig. 1A).

### The teaching method

The teaching method was structured into four steps, all delivered within a single day. Step 1 focused on familiarizing students with the direct ophthalmoscope (lasting 30 minutes); Step 2 covered proper patient and examiner positioning (lasting 10 minutes); Step 3 involved practical training using a photographic poster (lasting 10 minutes); and Step 4 required students to perform DF on each other (lasting 60 minutes). A detailed description of each step is provided below.

Step 1 Introduction to the direct ophthalmoscope (fundoscopy class)

This initial 30-minutes segment was designed to ensure that students were comfortable and proficient in using the direct ophthalmoscope. Each student was provided with a device and received explicit instruction on its operation. This included:

• Turning the ophthalmoscope on and off using the lateral button on the ophthalmoscope head.
• Adjusting the intensity of the light beam directed into the patient's eye.
• Manipulating the diopter wheel to achieve a clear focus on the internal structures of the eye. This adjustment is crucial for correcting refractive errors, such as myopia and hyperopia, in both the examiner and the patient.
• Selecting and utilizing different filters and light settings. For example, students learned that the green filter enhances the visualization of retinal nerve fiber layer defects and pale optic disc.

Furthermore, they have been taught to hold the ophthalmoscope with the hand ipsilateral to the eye being examined. The examiner should position the light exit face of the direct ophthalmoscope toward the patient, using the first, third, fourth, and fifth fingers to grasp the device's handle, while the second finger rests on the diopter wheel. The index finger is used to turn the device on and off (Fig. 1B).

Step 2 Patient and examiner positioning

This step, lasting 10-minutes, involved a demonstration by the instructor, with one student acting as the patient. Students were instructed on how to position the patient and themselves for optimal examination conditions.

**Fig. 2:**
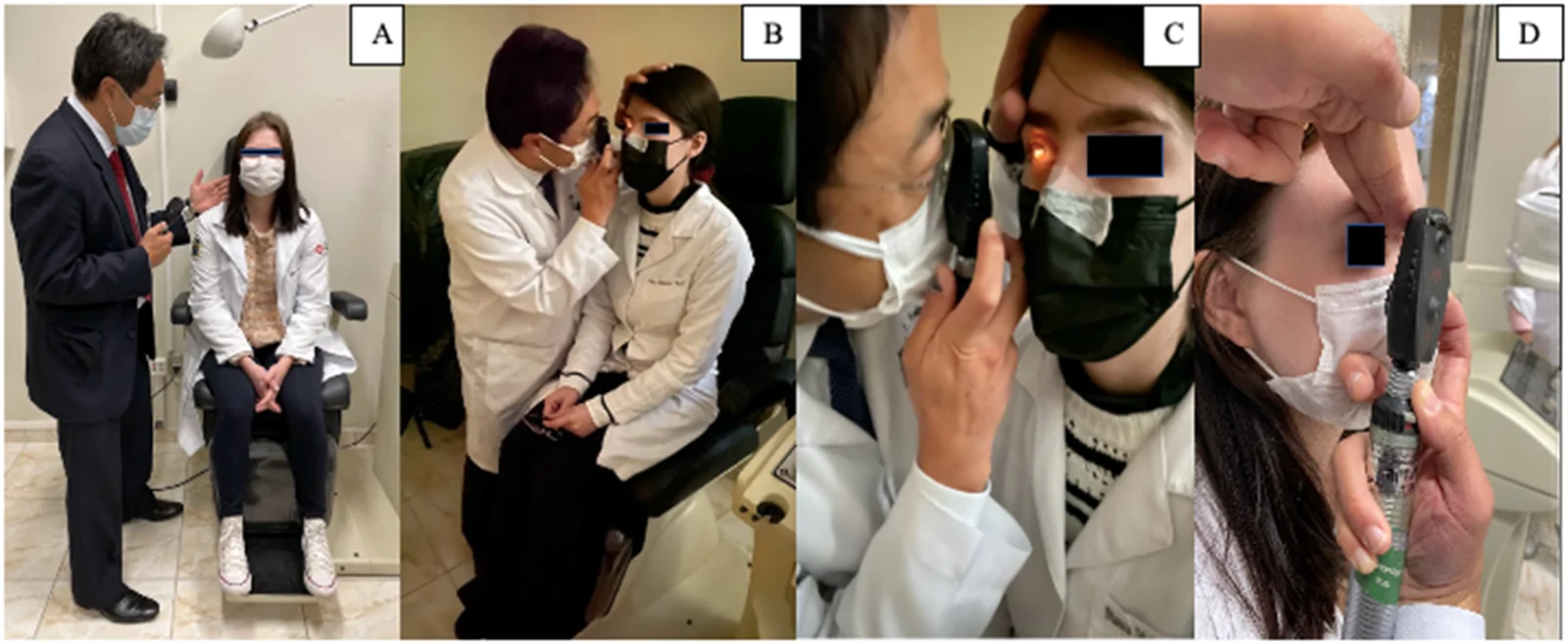
Patient and Examiner Positioning and Instrument Handling. (A) Doctor and patient initial positioning. Note the examiner’s “Judo base”, with body heading the patient, and proper examinee’s position, with the upper limbs relaxed alongside the body and legs closed. (B) Doctor’s approach towards the patient, with rotation of the torso laterally seeking for the red reflex. (C and D) Distance on which corneal reflex disappears, and it is possible to reach the eye fundus (approximately 2 fingerbreadths from the examinee’s eye). (D) Correct finger position on handling the ophthalmoscope. Source: The authors, 2022.
• Examiners are instructed to observe and follow the red reflex through the direct ophthalmoscope, a distinctive red spot that appears in the pupil when light reaches the retina. During the approach, the presence of a secondary white reflex, known as the corneal reflex, may also be noted, which results from light refracting off the patient's cornea. As the examiner approaches the patient (Fig. 2C) ‒ approximately two fingerbreadths from the face ‒ the corneal reflex diminishes, allowing assessment of the ocular fundus (Fig. 2D). Students were then encouraged to maintain this distance and adjust the diopter settings from zero to achieve optimal focus on optic disc. The latter is the point of reference of this technique, and once it is clear (correct myopia or hyperopia, if necessary, in the diopter wheel), there is no need to adjust the diopter knob further; if the patient is emmetropic, we found 0 diopters, without correction the optic disc is clearly visible in most patients.

• Patient Positioning: The patient should be seated comfortably with their head facing forward and their gaze fixed on a designated point, without wearing glasses. The patient's back should be supported by the chair, with their arms relaxed at their sides and their legs either together or crossed. The height of the patient's chair should be adjusted to align the patient's head with the examiner's, promoting ergonomic comfort for both individuals.
• Examiner Positioning: Students were instructed to observe the examiner's posture and approach to the patient. The examiner holds the ophthalmoscope to their eye and stands beside the patient, on the same side as the eye being examined. The examiner adopts a stance with feet shoulder-width apart for stability (the “Judo Base”) (Fig. 2A), and then rotates their torso laterally to establish a 90-degree angle with their base. The hand holding the ophthalmoscope should be ipsilateral to the patient's eye being examined, as should the examiner's eye used for the examination. The examiner's free hand is placed on the patient's forehead, with the thumb resting on the patient's brow (Fig. 2B). This positioning technique helps stabilize the examiner's approach and prevents them from getting too close to the patient's face, thereby increasing the student's confidence and ensuring patient comfort.

After the examination of the first eye, the positioning must be inverted to examine the second eye.

Step 3 Learning through the use of a photographic poster

**Fig. 3:**
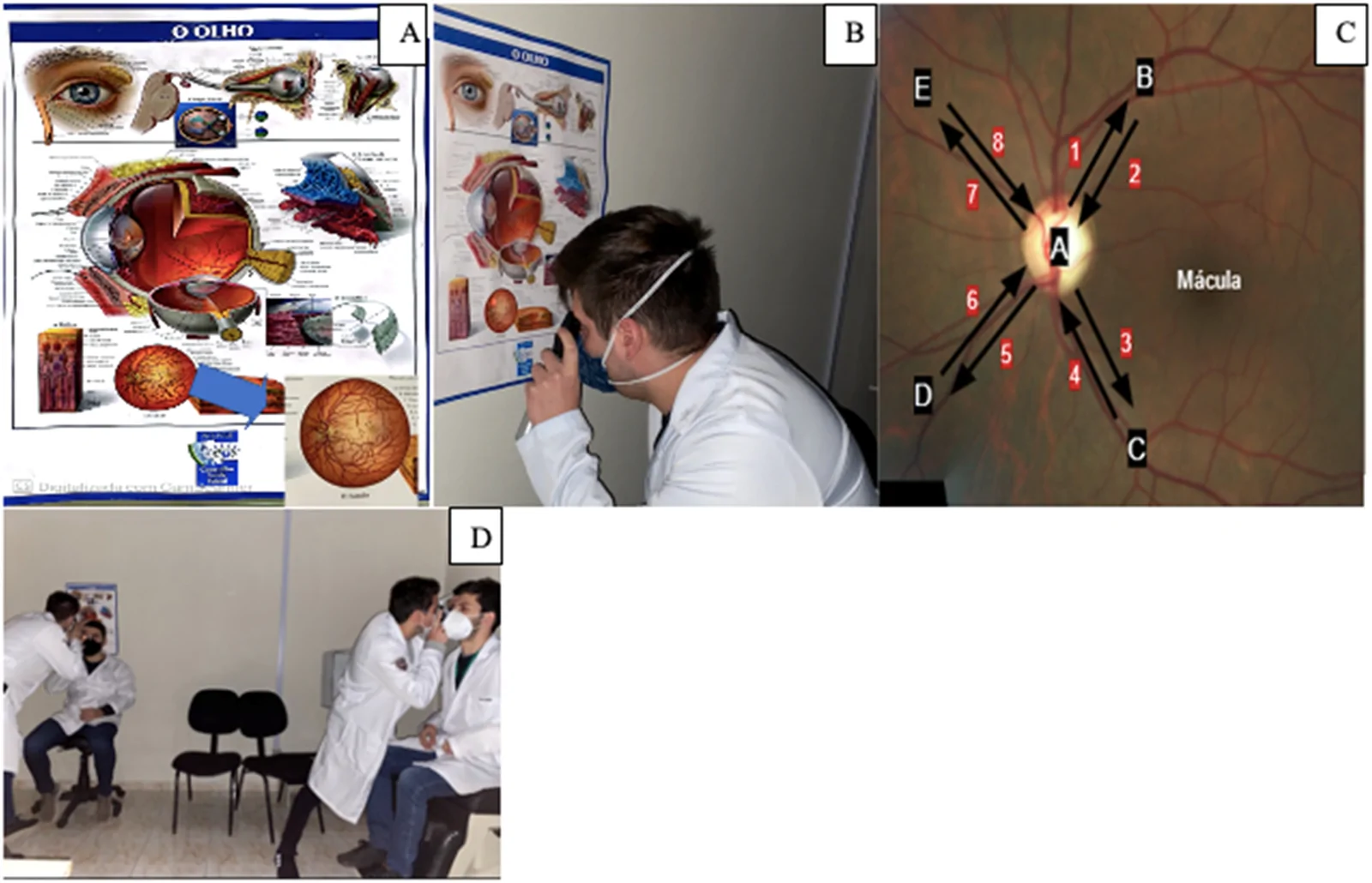
(A) The fundus photograph poster. (B) The students’ approach to the poster. (C) The correct order for fundus examination begins with section A (optic nerve head) and continues with the visualization of the quadrants, numbered from 1 to 8. (D) Students examining each other in pairs. Source: The Authors, 2022.

In this 10-minutes phase, students simulate the taught examination using a photographic poster of a fundus instead of a real eye (Fig. 3A). The pictures of this poster are not of the size of a real eye fundus, making the visualization much easier, but still provides a target for students to focus on with the direct ophthalmoscope.

As emphasized by the Cognitive Load Theory (CLT), the teaching of high-performance tasks can be facilitated by reducing either the extraneous or intrinsic cognitive load associated with the activity.^15^ In this context, when performing fundoscopic examination on a real patient, the learner is required to integrate multiple skills that have not yet been consolidated in working memory, executing them simultaneously.

These skills include developing the motor coordination necessary for proper positioning relative to the patient, handling the ophthalmoscope effectively, approaching the patient with confidence and without causing discomfort, and accurately identifying the structures of the ocular fundus.

To address this challenge, we propose reducing the intrinsic cognitive load of the task by dividing the learning process into two stages. In the first stage, the learner practices positioning and approach using an inanimate object (poster), thereby isolating these skills from the complexities of patient interaction and individual variability, reducing students’ anxiety. In the second stage, the examination is performed in a fully integrated manner, combining all required competencies. At this point, the overall cognitive load is reduced, as prior exposure to the instructional poster has already supported the partial internalization of these skills within working memory.

The students were given the following instructions: 1) Correctly hold the DO; 2) Correctly position the body relative to the examined eye; 3) Correctly approximate the patient’s eye; and 4) Practice correct posture and movement for visualizing the posterior pole.

Students were instructed to rotate their head and the direct ophthalmoscope together, to visualize fundus structures on the photographic poster. It was emphasized that the distance between this unit (head and ophthalmoscope) and the poster should remain constant throughout the simulation (Fig. 3B).

Instructions are given to students prior to the examination. The Optic Disc (OD) was identified as the initial structure to be visualized and correct error of refraction if needed, if the OD was not the first structure observed, students were advised to locate a vessel and follow it proximally, noting its increasing thickness as it approaches the disc area.

The overarching objective was to examine structures extending from the OD across all quadrants of the posterior pole. The recommended sequence began with the upper right quadrante with the left eye as example. After examining each quadrant, students were directed to return their focus to the OD as a central reference point before proceeding to the next quadrant in a clockwise direction. The final step involved a temporal shift of approximately two papillary diameters from the OD to assess the macular area (Fig. 3C).

Following the poster simulation, students were instructed to observe their colleagues.

Step 4 DF examination among students

In this 60-minutes segment, students were paired, with each individual taking turns as the examiner and the patient (Fig. 3D). After the initial examination, the roles were reversed.

In this step, students learn to visualize fundus structures, which is particularly challenging for beginners. Therefore, it is important that the lessons from the previous step are well internalized, a goal achieved through practice in front of the poster. As a result, during this phase of the method, students are better able to focus on and accurately observe structures in the fundus of a real eye.

For consistency, the right eye was examined first. The examination room was kept semi-dark to facilitate visualization of the fundus by minimizing pupillary constriction.

Examiners were instructed to set the DO to the smallest circle of light and adjust the focus to zero diopters. Prior to the examination, students acting as patients were instructed to keep both eyes open to avoid excessive muscle fatigue, as closing the contralateral eye requires increased muscular effort. The instructor was available to address any student questions.

### Questionnaire

All the initial 143 participants of the study answered the pre and post method questionnaire. The first part of it was administered before the direct fundus class to assess students' skills, challenges, and confidence in performing DF, particularly among those with prior experience. The second part of the questionnaire, distributed after the class, aimed to evaluate changes in confidence levels and perceived difficulties, thereby providing data to validate the teaching method's effectiveness.

The complete questionnaire used for both assessments is included as an Appendix.

## Results

### Prior to method application

Previous to method application, all 143 students were already exposed to direct ophthalmoscopy learning during other medical classes. Still, they reported various challenges in performing DF. The main difficulties included a lack of practical classes (72%), anxiety about distinguishing normal from pathological eye conditions (69%), difficulties in positioning themselves appropriately relative to the patient (67%), and insufficient theoretical preparation at home (32%). Furthermore, students expressed concerns about getting too close to patients, a lack of theoretical instruction, struggles with locating and identifying fundus structures, inadequate quality of DO limited training with the DO, and challenges related to examining non-dilated pupils.

### After method application

#### Students’ visualizations

The 143 students were directly asked which fundus structures they were able to recognize. Before the classes, 31.5% of the students (n = 45) reported being unable to visualize any fundus structures. After class, this percentage decreased to 1.4% (n = 2). Moreover, the proportion of students able to see at least one component of the posterior pole increased, from 68.5% (n = 98) to 98.6% (n = 141) following the intervention. This improvement was statistically significant p-value < 0.0001.

The assessment of students' ability to visualize different fundus structures included recognition of the retina, OD, OD cupping, OD margins, macula, fovea, retinal vessels, and differentiation between arteries and veins. Paired proportions were compared using McNemar’s test. Given the presence of multiple secondary outcomes, p-values were adjusted using the Benjamini-Hochberg false discovery rate procedure (q = 0.05). All results remained statistically significant after correction, except for the fovea assessment, which remains as a limitation of the study (Table 1).

**Table 1:** Visualized structures at DF before and after the training.

	Before	After	Chi-Square (χ^2^), and p-value after FDR^a^	Odds Ratio
**Total of students**	143	143		
	**N of students and (%)**	**N of students and (%)**		
**Visualize fundus**				
No	45 (31.5)	2 (1.4)		
Yes	98 (68.5)	141 (98.6)	39.2 (p < 0.0001)	96.54
**Identified optic disc**				
No	81 (56.6)	30 (21.0)		
Yes	62 (43.4)	113 (79.0)	39.68 (p < 0.0001)	9.49
**Identified disc cupping**				
No	105 (73.4)	76 (53.1)		
Yes	38 (26.6)	67 (46.9)	13.29 (p = 0.0003)	2.94
**Identified limits of optic disc**				
No	95 (66.4)	43 (30.1)		
Yes	48 (33.6)	100 (69.9)	36.13 (p < 0.0001)	6.17
**Identified macula**				
No	114 (79.7)	96 (67.1)		
Yes	29 (20.3)	47 (32.9)	5.78 (p = 0.018)	2.12
**Identified fovea**				
No	134 (93.7)	131 (91.6)		
Yes	9 (6.3)	12 (8.4)	0.24 (p = 0.62)	1.43
**Identified vessels**				
No	70 (49.0)	4 (2.8)		
Yes	73 (51.0)	139 (97.2)	62.13 (p < 0.0001)	66.69
**Differentiated veins from arteries**				
No	117 (8.8)	50 (35)		
Yes	26 (18.2)	93 (65)	52.48 (p < 0.0001)	9.39
**Differentiated right from left eye**				
No	133 (93.0)	79 (55.2)		
Yes	10 (7.0)	64 (44.8)	46.82 (p < 0.0001)	18.92

The statistical test used was Chi-Square MacNemar.

a p-values were corrected by Benjamini-Hochberg False Discovery Rate (FDR) procedure.

**Fig. 4:**
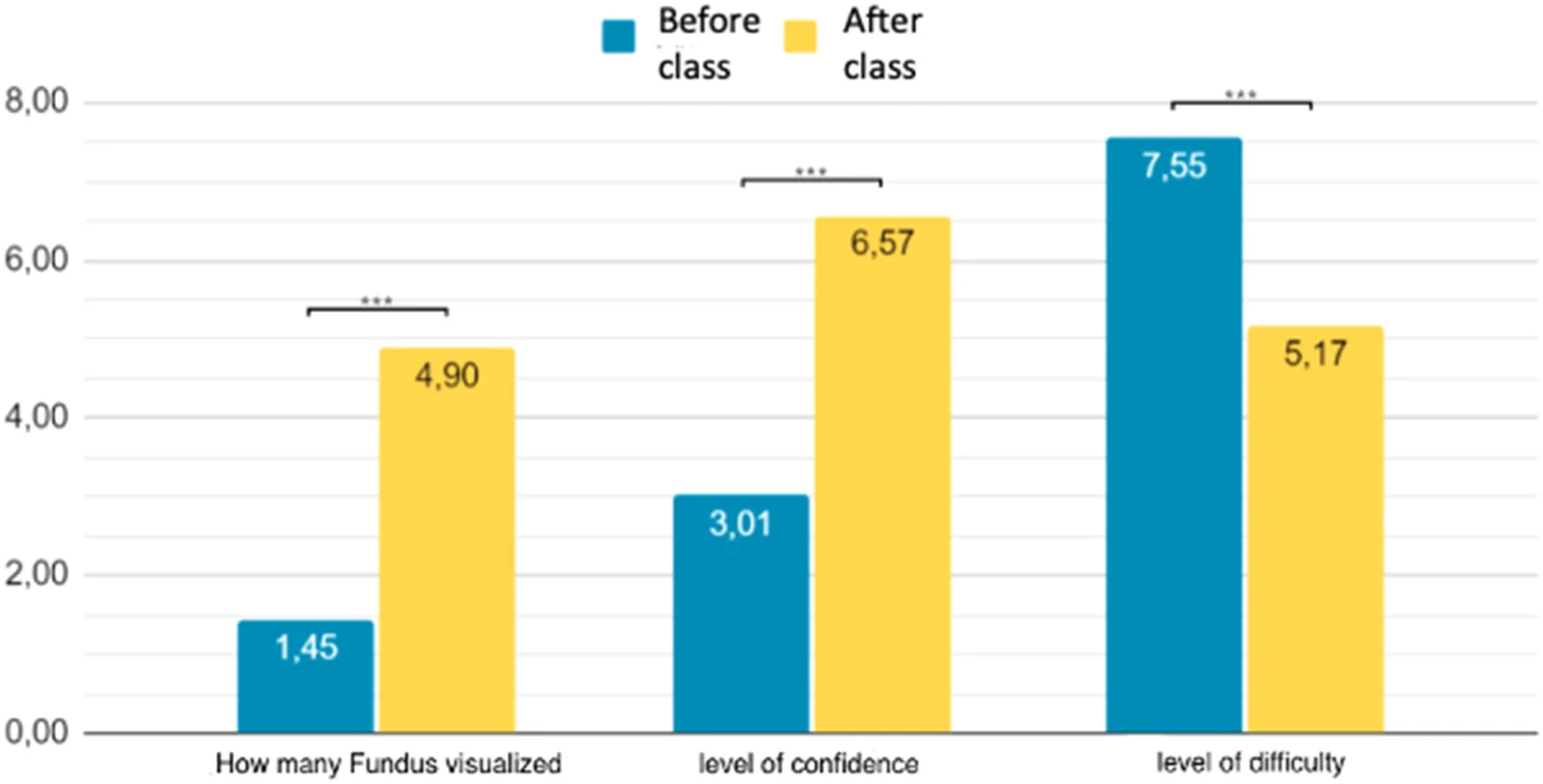
Comparison of results regarding the average of parameters: quantity of visualized fundus, level of student’s confidence and difficulty during DF before and after the training. *Wilcoxon Test (p < 0.001, effect size rank biserial correlation 0.92; 0.83; 0.87 respectively).

Student confidence increased, and the perceived level of difficulty decreased, both with statistical significance (p < 0.001) (Fig. 4).

## Discussion

The findings demonstrate that our method can be effectively learned, as evidenced by the 98.6% of students who successfully identified the fundus structures after the classes. The method enabled students to visualize key fundus structures, including the retina, OD, and blood vessels, with statistically significant improvement compared to pre-training data (p < 0.0001), except for the foveal area. The foveal area presents a challenge even for experienced clinicians, so it is understandable that beginner students find it particularly difficult to visualize. This difficulty may be attributed to its small size, which complicates examination of non-dilated pupils, as well as its high sensitivity to light, which can cause patient discomfort and resistance during the procedure. It also stimulates further pupil constriction making it harder to perform fundoscopy. This limitation highlights the need for further refinement of the method in future iterations, such as implementing pupil dilation or increasing the number of classes given following the methodology, which improve CLT.

Previous studies by Samaranayake et al.^9^ and Schulz and Hodgkins^16^ identified insufficient practical classes and improper handling of the DO as major challenges in learning fundoscopy. The present study addressed these issues by incorporating theoretical instructions before practical sessions and extending the practical training duration to 60-minutes. The observed increase in students’ confidence and reduction in perceived difficulty in performing the examination further support the effectiveness of this new DF learning method.

Key components of the teaching technique align with the TOTems Study^17^ and the systematic approach to examining internal eye structures detailed in “Oftalmologia Prática”,^18^ but they lack in explaining details of the correct doctor´s approach and positioning. There is no report of such detailed description of body movements as our methodology does, such as torso rotation towards the patient, and the guidance of the exam based on the corneal reflex. This innovative training presents a comprehensive methodology that focuses on critical elements of effective examination performance, including proper interpersonal positioning, mutual respect, and strategies for patient engagement throughout the examination process.

### Study limitations

The study's findings may be limited by potential response bias due to the subjective nature of student questionnaire answers. In addition, the wording of the post-methodology questionnaire may have guided students' responses, potentially introducing acquiescence bias. The possibility of students providing favorable feedback to meet perceived expectations exists and may also be a confounding factor. Additionally, we must emphasize the limitations of students' self-reported visualization, which may not correlate with technically accurate performance.

The assessment of students’ learning outcomes could be reconsidered by incorporating more clinically oriented evaluation methods. For instance, students could be asked to match fundus photographs to the corresponding patient cases or to reproduce, through schematic drawings, the retinal findings observed during the examination. Such approaches may provide a more comprehensive assessment of both diagnostic recognition and observational skills.

Furthermore, additional statistical analyses could be conducted to investigate the influence of potential confounding variables, including the presence of refractive errors among students and their previous exposure to direct ophthalmoscopy training. Evaluating these factors may contribute to a more accurate interpretation of the educational intervention’s effectiveness.

The cross-sectional design of the survey also limits the assessment of long-term retention of DF proficiency. It is fair to admit further reviews techniques to ensure the data results. Future studies should also incorporate an appropriate control group to strengthen the study design.

Furthermore, foveal examination remains a challenge in fundoscopy training, highlighting the need for further methodological improvements in the future.

## Conclusion

This study presents a novel methodology designed to enhance the learning of DF without requiring pupil dilation. The primary objective is to equip medical students with the ability to recognize appropriate clinical contexts for DF, in an effort to ensure its effective application across various medical specialties, including ophthalmology, neurology, and general medicine, even in clinics with lack of technological equipment’s. The study also advocates for universities to reconsider curricular strategies to incorporate this methodology, fostering greater confidence and proficiency in clinical practice among graduates. The results of this study suggest the effectiveness of the proposed methodology in teaching students to perform direct fundoscopy.

## Declaration of generative AI and AI-assisted technologies in the manuscript preparation process

During the preparation of this work the author(s) used Google Gemini and CHAT GPT in order to correct grammar mistakes and to enhance clarity and coherence. After using this tool/service, the author(s) reviewed and edited the content as needed and take(s) full responsibility for the content of the published article.

## Abbreviations

DF, Direct Fundoscopy; DO, Direct Ophthalmoscope; UFPR, Universidade Federal do Parana.

## Ethical issues

The study was conducted in accordance with the ethical principles outlined in the Declaration of Helsinki, and all participants provided written informed consent prior to their inclusion. Ethics Committee study protocol number: CAAE 24561313.3.0000.0096.

## Study guidelines

This study follows the STROBE statement for observational studies.

## Fundings

This research did not receive any specific grant from funding agencies in the public, commercial, or not-for-profit sectors.

## CRediT authorship contribution statement

**Aline Shiokawa:** Conceptualization, Investigation, Methodology, Writing – review & editing. **Mario L.R. Monteiro:** Writing – review & editing. **Raffael Massuda:** Writing – review & editing. **Marcelo Carriello:** Writing – review & editing. **Ariane B. Fiorentin:** Writing – original draft, Data curation, Investigation. **Max V. Schramm:** Writing – original draft, Data curation, Investigation. **Mario T. Sato:** Conceptualization, Methodology, Data curation.

## Declaration of competing interest

The authors declare no conflicts of interest.

## Data Availability

The datasets generated and/or analyzed during the current study are available from the corresponding author upon reasonable request.

## Appendix Supplementary materials

Image, application 1

Image, application 2
