# Supplementary material for: A new teaching method of direct fundoscopy for daily clinical practice
Source: Clinics (Sao Paulo). 2026 Sep 5;81:101085. doi: 10.1016/j.clinsp.2026.101085 (PMC13571928; doi:10.1016/j.clinsp.2026.101085)
Supplement: Supplementary file 2 [file mmc2.docx]

CLINICS-D-25-01799_Supplementary Material

**Appendix** Questionary.

**Before today’s class**

1. At the Direct Fundoscopy (DF) exams that you’ve already performed, have ever seen an optic disc?

Yes (  ) No (  )

If “yes”, how many times?

If “yes”, DF wa performed with pupil dilation?

( ) Yes. ( ) No

If “yes”, have you distinguished the structures? (Mark an X the structures seen):

(  ) Retina; (  ) Optic disc; (  ) Optic disc cupping; (  ) Limits of optic disc; (  ) Macula;  (  ) Fovea

Vessels: (  ) Trajectory; (  ) Differentiation between vessels and arteries; (  ) Differentiation between right and left optic disco (looking for asymmetries)

2. In which discipline of the course have you had contact with fundoscopy?

3. In which of them have you been able to see de fundus?

4. From zero to ten, what was the level of confidence to perform DF in a random patient?

5. From zero to ten, what was the level of difficulty to perform DF in a random patient?

6. Which were the difficulties found?

(  ) Motor coordination

(  ) Fear of getting near the patient

(  ) Fear of not identifying what is normal and what is not on fundus.

(  ) Difficulty of positioning towards the

(  ) Insufficient theorical classes

(  ) Insufficient practical classes

(  ) Insufficient own study/ review of theorical classes

Others:

7. do you have any visual issue? If YES, which one and how many diopters?

Today’s class: Simultion training

**Beginning time**

Ending time

Did you feel tired during the long period training?

(  ) Yes  (  ) No

If “yes”, at what time, approximately, have you felt tired?

**After today’s class**

Did you manage to see optic disc?

Yes (  ); No (  )

8. If YES, how many have you were able to see?

9. Structures you’ve seen today:

(  ) Retina; (  ) Optic disc; (  ) Optic disc cupping; (  ) Limits of optic disc; (  ) Macula;  (  ) Fovea; Vessels: (  ) Trajectory; (  ) Differentiation between vessels and arteries; (  ) Differentiation between right and left optic disco (looking for asymmetries)

10. Which are the method’s potentials?

(  ) Having theorical acclimatation previously to the practical class

(  ) Acclimatation with ophthalmoscope use

(  ) Training primarily with illustration

(  ) Training DF for more than 30-minutes

(  ) Training DF for more than 60-minutes

(  ) Training with the colleague and not the patient

(  ) Training in more than one person

(  ) Seek the red reflex

11. Do you believe that this practical training for long period practical class, as did today, created good methodology fixation?

Yes (  ) No (  )

12. After the end of the class, from 0 to 10, how is your confidence in examining a random patient’s eye?

13. After the end of the class, from 0 to 10, how is your difficulty in examining a random patient’s eye?

14. Is there something you would recommend for the improvement of this methodology?
